# Declining semen quality among south Indian infertile men: A retrospective study

**DOI:** 10.4103/0974-1208.38972

**Published:** 2008

**Authors:** Adiga SK, Jayaraman V, Kalthur G, Upadhya D, Kumar P

**Affiliations:** Division of Reproductive Medicine, Kasturba Medical College, Manipal University, Manipal, India

**Keywords:** Azoospermia, infertility, reproductive function, semen quality, seminal parameters, sperm concentration, sperm decline, spermatozoa

## Abstract

**BACKGROUND:**

Male reproductive function has recently attracted increasing attention due to reports on time-related decline in semen quality. Furthermore, regional differences in the semen quality have also been reported.

**AIM:**

To investigate the semen quality among large cohort of infertile individuals at a regional level, in terms of the sperm concentration, total sperm motility, sperm morphology and incidence of azoospermia over a period of 13 years.

**SETTING:**

University infertility clinic at Kasturba Hospital, Manipal which is a tertiary healthcare centre serving the general population.

**DESIGN:**

Retrospective analysis.

**MATERIALS AND METHODS:**

This includes a total of 7770 subjects who presented for semen analysis from 1993 to 2005. The data regarding ejaculate volume, sperm density, motility, morphology and the incidence of azoospermia were collected.

**STATISTICAL ANALYSIS USED:**

One way analysis of variance (ANOVA), regression analysis and Chi square analysis.

**RESULTS:**

The average sperm density among infertile men during 2004-2005 was 26.61 ± 0.71 millions/mL which was significantly lower than the average sperm density observed in 1993-1994 (38.18 ± 1.46 millions/mL). Similar trend was also observed for sperm motility (47.14% motile sperms *vs.* 61.16%) and normal sperm morphology (19.75% *vs.* 40.51%). Interestingly, the incidence of severe oligospermia (mean sperm density <10 millions/mL) observed in 2002-2005 and 1993-1997 demonstrated a significant inverse relationship (*P* < 0.001).

**CONCLUSION:**

Our study provides the first evidence that the quality of human semen evaluated for infertility is deteriorating in the southern part of the India over the years, probably due to environmental, nutritional, life style or socioeconomic causes.

Concerns over a global decline in sperm quality have attracted the attention of the scientific community and public alike. Time and over again, numerous studies have been published, supporting a compromise in sperm quality or dismissing the same.[1–5] Although, the reason for this remains unclear, it is suggested that environmental factors are mainly responsible for alteration in the semen quality.[6,7]

Analysis of retrospective data indicates that sperm counts may have declined in some parts of the world, but there seem to be geographical variations in the semen quality.[8–10] The reason for geographic variations in semen characteristics is unclear but may be due to environmental, nutritional, socioeconomic or other unknown causes.[11] Although, the baseline semen quality and sperm functional parameters in fertile Indian men have been documented,[12] the first report investigating changes in semen characteristics in Indian men over the period of time demonstrated no significant decline in sperm count from 1990 to 2000.[4] In view of high-population density, heterogeneous nature of the Indian population, climatic differences and dietary habits, it is necessary to know whether similar trend exists within the different parts of the same country. The aim of this study is to investigate the semen quality among South-Indian men seeking infertility evaluation, in terms of the sperm concentration, total sperm motility, sperm morphology and incidence of azoospermia at a regional level over a period of 13 years.

## MATERIALS AND METHODS

The data for the present analysis were obtained from patients visiting university infertility clinic at Kasturba Hospital, Manipal which is a tertiary healthcare centre serving the general population mainly from three states of the country, namely Karnataka, Kerala and Goa. The study was based on the retrospective review of a total of 7770 subjects, who had their semen examined as a part of the infertility evaluation, at the clinic, between the years 1993 and 2005. All patients were asked to provide semen sample after 3-5 days of ejaculatory abstinence. Semen specimens were produced by masturbation directly into a sterile plastic container, in a room specially provided for this purpose and located adjacent to the laboratory. After liquefaction, semen processing and analysis was performed according to the World Health Organization recommendations.[13,14] Seminal volume was determined in a graduated tube. During the entire period of study, sperm concentration was assessed by conventional method using Makler counting chamber (Sefi Medical Instruments, Israel) and expressed in millions/mL. The sperm motility was assessed in at least 100 sperms and expressed as percent of motile sperm (sum of rapid progression plus slow progression sperm).[13] Sperm morphology was assessed by Leishmen's stain from 1993 to 1998 and by Eosin-Nigrosin stain from 1999 to 2005. Basic descriptive statistics (means ± standard error) were calculated for the study groups. Statistical analysis of the means between different study periods was performed using one-way analysis of variance (ANOVA) for normality distribution. Regression analysis was carried out using Statistical Package for the Social Sciences (SPSS) statistical package. Chi-square test was performed for analyzing the azoospermia incidence. A *P*-value < 0.05 was considered statistically significant.

## RESULTS

**Figure 1:**
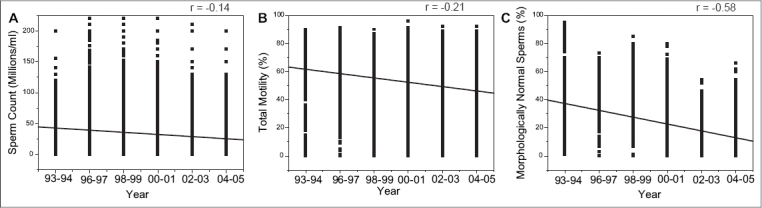
The linear regression analysis between the study period and (A) sperm count, (B) total sperm motility and (C) sperm morphology

The descriptions of seminal parameters for all the 7770 participants are presented in Table 1. The data showed statistically significant differences in the seminal characteristics of the subjects analyzed between the time gap of 13 years, i.e., 1993-1994 and 2004-2005 most notably in the sperm density, motility and normal sperm morphology, assessed according to the WHO criteria. The mean sperm density among infertile men during 2004-2005 was 26.61 ± 0.71, which was significantly lower than the average sperm density observed in 1993-1994 (38.18 ± 1.46 millions/mL). Similar trend was observed for sperm motility (47.14% motile sperms *vs.* 61.16%) and also sperm morphological abnormalities (19.75% normal forms *vs.* 40.51%). The results of the regression analyses exploring the relationship between semen parameters and time period are presented in Figure 1. The results clearly demonstrated an inverse (linear) association with count (*r* = −0.14339), motility (*r* = −0.20505) and morphology (*r* = −0.58372), suggesting a consistent time-related decline in the semen quality.

**Table 1:** Semen parameters in infertile men from 1993 to 2005

	Year					
	
	1993-1994	1996-1997	1998-1999	2000-2001	2002-2003	2004-2005
*N*	589	738	1260	2050	1523	1610
Seminal volume (ml)	1.707 ± 0.04	2.076 ± 0.05	2.06 ± 0.03	2.05 ± 0.02	2.44 ± 0.03	2.64 ± 0.03
Sperm count (millions/ml)	38.18 ± 1.46	40.26 ± 1.55	37.78 ± 1.13	35.04 ± 0.89	23.64 ± 0.70	26.61 ± 0.71
Total sperm motility (%)	61.16 ± 0.93	61.15 ± 0.58	54.74 ± 0.68	51.98 ± 0.52	48.70 ± 0.57	47.14 ± 0.54
Sperms with normal morphology (%)	40.51 ± 0.86	42.87 ± 0.64	24.71 ± 0.61	17.36 ± 0.38	14.44 ± 0.28	19.75 ± 0.33
Incidence of azoospermia (%)	5.942	7.994	8.4126	8.3414	8.0105	7.2670

**Figure 2:**
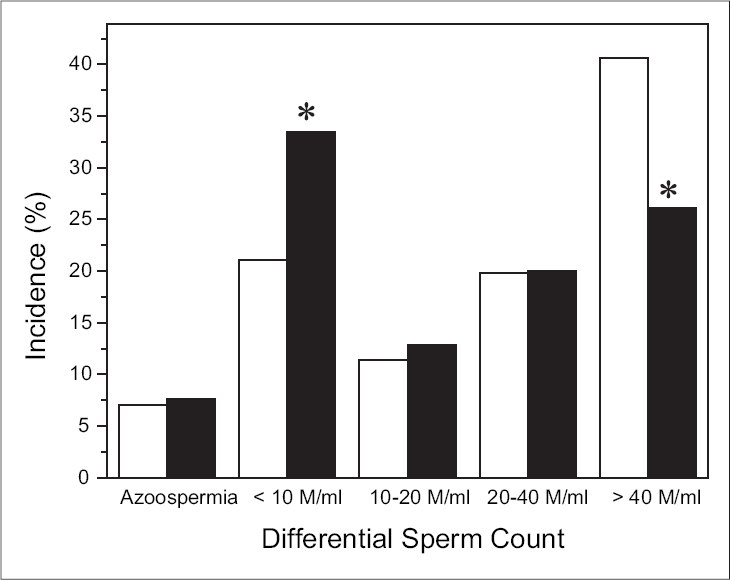
The incidence of azoospermia and differential sperm count between □ 1993-97 and ■ 2002-2005. *P < 0.001 with respective group

Most noticeably, there was a significant increase in the number of patients with severe oligospermia (sperm count < 10 millions/mL) in 2002-2005 when compared to 1993-1997. In contrast, the number of subjects having a mean sperm count >40 millions sperm/mL reduced significantly in 2002-2005 when compared to 1993-1997 (26.07% *vs.* 40.61%), showing an inverse relationship. However, no significant difference in the incidence was observed for the sperm count ranging from 20 to 40 millions/mL [Figure 2]. Similarly, there was no significant difference in the incidence of severe asthenospermia between 1993-1997 and 2002-2005 (data not shown). A marked but non-significant increase was noted in the volume of the semen and the incidence of azoospermia between 1993-1994 and 2004-2005 [Table 1].

## DISCUSSION

This is the first study to analyze the semen quality in large group of Indian men and our data clearly illustrates that quality of human semen is deteriorating over the period of 13 years. Our findings support previous reports that the quality of human semen seems to be declining in other parts of the world.[1–2,15–16] In particular, the decline in sperm count was 30.31% where as sperm motility and morphology was reduced by 22.92% and 51.25%, respectively, between the time span of 13 years. Furthermore, the regression analysis also confirmed a true decline in the semen quality over this period. During the period of study, there were very few changes in the techniques and personnel involved in the analysis of semen. Technicians adhered to strict quality control and the equipments used were same throughout the entire study period.

The decline in the semen quality coincides with an increasing incidence of abnormalities of the male genital tract including testicular cancer and cryptorchidism in various countries.[17,18] More importantly, the increase in the incidence of sperm morphological abnormalities in addition to low-sperm count observed in this study indicates qualitative impairment of spermatogenesis and perhaps of the Sertoli cells.[19] Since the criteria to assess morphologically normal sperm has been modified in our centre from 1999 where all the borderline abnormalities were considered as abnormal, this could have caused a sudden decline in the percentage of morphologically normal sperm from 1999 to 2000 onwards.

The reason for decline in semen quality is possibly due to environmental, nutritional, socioeconomic or other unknown causes. [1–2,9,11,20] *In utero* exposures to exogenous estrogenic compounds are capable of altering neonatal testicular development and reducing sperm production in adult men.[19–22] Diethylstilbestrol is thought to be responsible for an increase in abnormalities of reproductive tract and for reduction in the output and fertilizing potential of sperm of male offspring.[23]

In view of substantial geographical and ethnic variation in semen quality reported by several investigators, [9,12,24–27] it is necessary to investigate population-based trends in semen quality over time. Earlier studies indicated decline in the semen quality in some parts of the world, but there seem to be geographical variations in the time-related decline in semen quality.[9–11,27–30] The baseline sperm concentration and motility for Indian men was reported as 68.22 ± 15.14 millions/mL and 40.95 ± 9.15%, respectively[12] and a previous study failed to demonstrate any change in the semen quality among infertile men in the northern part of the India for a period of 11 years.[4] In contrast, the mean sperm count observed in our study was 26.61 millions/mL which was much lower than the above-mentioned baseline value of 68.22 millions/mL for Indian men.[12] However, there was no significant difference in the motility between two studies, suggesting a definite decline in the sperm concentration in the southern part of the India.

The present study has several limitations. (1) The subjects included here were infertile patients and do not represent the general population, (2) no data are available on the factors affecting semen quality such as occupation of the subjects, smoking, food habits and level of stress and (3) the duration of study is only 13 years which may be too short to come to any conclusion. However, longer study period may be influenced by many factors like change in laboratory staff, equipments, methodology over the period of time.

India has high heterogeneous human population density contributing to approximately 25% of the global population. Due to diverse environmental, nutritional and socioeconomic factors and climate conditions, it is important to assess the semen quality in different parts of the country for further validation of the statement. The significant time-related decline in semen quality observed in this study has important implications with respect to fertility and further studies using large cohort of normal subjects with additional information on their occupation, socioeconomic condition, life-style related factors are warranted in order to confirm the findings of current report.
